# Supplementary material for: The lead and cadmium content in rice and risk to human health in China: A systematic review and meta-analysis
Source: PLoS One. 2022 Dec 15;17(12):e0278686. doi: 10.1371/journal.pone.0278686 (PMC9754602; doi:10.1371/journal.pone.0278686)
Supplement: S1 Fig — (DOCX) [file pone.0278686.s001.docx]

**S1 Fig. Sensitivity analysis**

**Figure 1. The sensitivity analysis of Pb (given named study was omitted).**

 **Figure 2. The sensitivity analysis of Cd (given named study was omitted).**
