# Supplementary material for: The lead and cadmium content in rice and risk to human health in China: A systematic review and meta-analysis
Source: PLoS One. 2022 Dec 15;17(12):e0278686. doi: 10.1371/journal.pone.0278686 (PMC9754602; doi:10.1371/journal.pone.0278686)
Supplement: Attachment — Submitted filename: PONE-D-22-16207_reviewer.pdf [file pone.0278686.s002.pdf]

PLOS ONE

Heavy metals in rice and risk to human health in China: a systematic review and meta-analysis

--Manuscript Draft--

**Manuscript Number:**	PONE-D-22-16207
**Article Type:**	Research Article
**Full Title:**	Heavy metals in rice and risk to human health in China: a systematic review and meta-analysis
**Short Title:**	Heavy metals in rice and risk to human health in China
**Corresponding Author:**	HONGYU ZHOU<br>Chongqing Medical University<br>Chongqing, CHINA
**Keywords:**	rice; Heavy Metals; lead; cadmium; health risk; China; meta-analysis
**Abstract:**	Numerous studies have investigated concentrations of lead (Pb) and cadmium (Cd) in rice in China, but have come to divergent conclusions. Therefore we systematically reviewed and meta-analyzed the available evidence on levels of Pb and Cd in rice in different regions of China in order to assess the potential risk to human health. The meta-analysis included 24 studies of Pb levels and 29 studies of Cd levels, published in 2011-2021. The pooled Pb concentration in rice was 0.10 mg per kg dry weight (95% CI 0.08−0.11), while the pooled Cd concentration was 0.16 mg per kg dry weight (95% CI 0.14−0.18). These levels are within the limits specified by national food safety standards. However, the total target hazard quotient for both metals exceeded 1.0 for adults and children, suggesting that rice consumption poses a health risk.
**Order of Authors:**	XIANLIANG HUANG, Postgraduate
	BO ZHAO, Postgraduate
	YANLEI WU, Postgraduate
	MINGTIAN TAN, Postgraduate
	LISHA SHEN, Undergraduate
	GUIRONG FENG, Postgraduate
	XIAOSHAN YANG, Doctor
	SHIQI CHEN, Undergraduate
	HONGYU ZHOU, Doctor
**Additional Information:**	
**Question**	**Response**
**Financial Disclosure**<br><br>Enter a financial disclosure statement that describes the sources of funding for the work included in this submission. Review the submission guidelines for detailed requirements. View published research articles from *PLOS ONE* for specific examples.<br><br>This statement is required for submission and **will appear in the published article** if the submission is accepted. Please make	This work was supported by the National Key R&D Program of China and Chongqing Science and Technology Bureau.

sure it is accurate.<br><br>**Unfunded studies**<br>Enter: *The author(s) received no specific funding for this work.*<br><br>**Funded studies**<br>Enter a statement with the following details:<br>• Initials of the authors who received each award<br>• Grant numbers awarded to each author<br>• The full name of each funder<br>• URL of each funder website<br>• Did the sponsors or funders play any role in the study design, data collection and analysis, decision to publish, or preparation of the manuscript?<br>• **NO** - Include this sentence at the end of your statement: *The funders had no role in study design, data collection and analysis, decision to publish, or preparation of the manuscript.*<br>• **YES** - Specify the role(s) played.<br><br>* typeset	
**Competing Interests**<br><br>Use the instructions below to enter a competing interest statement for this submission. On behalf of all authors, disclose any competing interests that could be perceived to bias this work—acknowledging all financial support and any other relevant financial or non-financial competing interests.<br><br>This statement is required for submission and **will appear in the published article** if the submission is accepted. Please make sure it is accurate and that any funding sources listed in your Funding Information later in the submission form are also declared in your Financial Disclosure statement.<br><br>View published research articles from *PLOS ONE* for specific examples.	The authors have declared that no competing interests exist.

**NO authors have competing interests**<br><br>Enter: *The authors have declared that no competing interests exist.*<br><br>**Authors with competing interests**<br><br>Enter competing interest details beginning with this statement:<br><br>*I have read the journal's policy and the authors of this manuscript have the following competing interests: [insert competing interests here]*<br><br>* typeset	
**Ethics Statement**<br><br>Enter an ethics statement for this submission. This statement is required if the study involved:<br><br>• Human participants<br>• Human specimens or tissue<br>• Vertebrate animals or cephalopods<br>• Vertebrate embryos or tissues<br>• Field research<br><br>Write "N/A" if the submission does not require an ethics statement.<br><br>General guidance is provided below. Consult the submission guidelines for detailed instructions. **Make sure that all information entered here is included in the Methods section of the manuscript.**	N/A

Powered by Editorial Manager® and ProduXion Manager® from Aries Systems Corporation

**Format for specific study types**<br><br>**Human Subject Research (involving human participants and/or tissue)**<br>• Give the name of the institutional review board or ethics committee that approved the study<br>• Include the approval number and/or a statement indicating approval of this research<br>• Indicate the form of consent obtained (written/oral) or the reason that consent was not obtained (e.g. the data were analyzed anonymously)<br><br>**Animal Research (involving vertebrate animals, embryos or tissues)**<br>• Provide the name of the Institutional Animal Care and Use Committee (IACUC) or other relevant ethics board that reviewed the study protocol, and indicate whether they approved this research or granted a formal waiver of ethical approval<br>• Include an approval number if one was obtained<br>• If the study involved *non-human primates*, add *additional details* about animal welfare and steps taken to ameliorate suffering<br>• If anesthesia, euthanasia, or any kind of animal sacrifice is part of the study, include briefly which substances and/or methods were applied<br><br>**Field Research**<br><br>Include the following details if this study involves the collection of plant, animal, or other materials from a natural setting:<br>• Field permit number<br>• Name of the institution or relevant body that granted permission	
**Data Availability**<br><br>Authors are required to make all data underlying the findings described fully available, without restriction, and from the time of publication. PLOS allows rare exceptions to address legal and ethical concerns. See the PLOS Data Policy and FAQ for detailed information.	Yes - all data are fully available without restriction

A Data Availability Statement describing where the data can be found is required at submission. Your answers to this question constitute the Data Availability Statement and **will be published in the article**, if accepted.<br><br>**Important:** Stating 'data available on request from the author' is not sufficient. If your data are only available upon request, select 'No' for the first question and explain your exceptional situation in the text box.<br><br>Do the authors confirm that all data underlying the findings described in their manuscript are fully available without restriction?	
**Describe where the data may be found in full sentences. If you are copying our sample text, replace any instances of XXX with the appropriate details.**<br><br>• If the data are **held or will be held in a public repository**, include URLs, accession numbers or DOIs. If this information will only be available after acceptance, indicate this by ticking the box below. For example: *All XXX files are available from the XXX database (accession number(s) XXX, XXX.).*<br>• If the data are all contained **within the manuscript and/or Supporting Information files**, enter the following: *All relevant data are within the manuscript and its Supporting Information files.*<br>• If neither of these applies but you are able to provide **details of access elsewhere**, with or without limitations, please do so. For example:<br><br>*Data cannot be shared publicly because of [XXX]. Data are available from the XXX Institutional Data Access / Ethics Committee (contact via XXX) for researchers who meet the criteria for access to confidential data.*<br><br>*The data underlying the results presented in the study are available from (include the name of the third party*	All relevant data are within the manuscript and its Supporting Information files.

and contact information or URL).<br>• This text is appropriate if the data are owned by a third party and authors do not have permission to share the data.<br><br>* typeset	
Additional data availability information:	Tick here if the URLs/accession numbers/DOIs will be available only after acceptance of the manuscript for publication so that we can ensure their inclusion before publication.

Manuscript Click here to access/download;Manuscript;Manuscript.docx

Heavy metals in rice and risk to human health in China: a systematic review and meta-analysis

Xianliang Huang[a,b], Bo Zhao[a,b], Yanlei Wu[a,b], Mingtian Tan[a,b], Lisha Shen[c], Guirong Feng[a,b], Xiaoshan Yang[a], Shiqi Chen[a,b], Hongyu Zhou[d,e*]

[a] Food Laboratory, Chongqing Institute for Food and Drug Control, Chongqing 401121, China

[b] Key Laboratory of Condiment Supervision Technology for State Market Regulation, Chongqing 401121, China

[c] Chongqing Academy of Chinese Materia Medica, Chongqing 400065, China

[d] College of Public Health and Management, Chongqing Medical University, Chongqing 400016, China

[e] The First Affiliated Hospital of Chongqing Medical University, Chongqing 400016, China

* Corresponding author

Address: No.1 Yixueyuan Road, Yuzhong District, Chongqing 400016, China

Tel.: +86 23 8901 2364

Fax: +86 23 8901 2364

E-mail address: hyzhou@cqmu.edu.cn

Abstract

Numerous studies have investigated concentrations of lead (Pb) and cadmium (Cd) in rice in China, but have come to divergent conclusions. Therefore we systematically reviewed and meta-analyzed the available evidence on levels of Pb and Cd in rice in different regions of China in order to assess the potential risk to human health. The meta-analysis included 24 studies of Pb levels and 29 studies of Cd levels, published in 2011-2021. The pooled Pb concentration in rice was 0.10 mg per kg dry weight (95% CI 0.08−0.11), while the pooled Cd concentration was 0.16 mg per kg dry weight (95% CI 0.14−0.18). These levels are within the limits specified by national food safety standards. However, the total target hazard quotient for both metals exceeded 1.0 for adults and children, suggesting that rice consumption poses a health risk.

Keywords

rice; heavy metals; lead; cadmium; health risk; China; meta-analysis

Introduction

Rice is a well-known staple food, consumed by about 50% of the population in more than 100 countries around the world. As the most populous country in the world, China is the largest producer and consumer of rice in the world. China's annual rice production totals approximately 2.07 × 10^{11} kg and accounts for nearly 34% of total global output (1-3). Growing industrialization and use of fertilizer have led to the continuing accumulation of toxic heavy metals in the soil of rice paddies, from which the metals can enter rice (4, 5). This accumulation is especially high in southern China, which has rapidly industrialized (6).

Many studies have shown that the heavy metal content in rice exceeds food safety standards in China (7), especially levels of cadium (Cd) and and lead (Pb) (8-10). The legal limit for both metals in rice is 0.2 mg/kg. The mean Cd levels in rice grain have been reported to be 0.69 mg/kg in Xiangtan County of Hunan Province (11), 0.62 mg/kg in Shaoguan City of Guangdong Province (12), and 0.29 mg/kg along the Yangtze River in Hubei, Hunan, and Jiangxi Provinces (13). The study in the Yangtze River area has also reported a mean Pb level of 0.25 mg/kg in rice grain (13).

Elevated dietary consumption of Pb and Cd from rice may harm human health (14, 15). Cd can damage kidneys as well as the pulmonary, cardiovascular, and musculoskeletal systems. Elevated Cd consumption has also been linked to Itai-Itai symptom (16-19).

Pb, for its part, can damage the immune, digestive, and nervous systems, as well as compromise cognitive development (20-22). Several studies in different regions of China have assessed whether levels of Pb and Cd in rice pose a health risk (23-25), but they have come to divergent conclusions. For example, a study in Guizhou Province concluded that levels of Cd and Pb in rice were too low to pose a health risk (26), while a study in the Pearl River Delta concluded the opposite (27). The relatively small samples in individual studies has prevented a coherent, overall evaluation of risk.

Therefore we systematically reviewed and meta-analyzed the available evidence on levels of Pb and Cd in rice in different regions of China and assessed the risk to human health.

Materials and methods

Search strategy

Two authors (B.Z. and G.R.F.) searched for relevant studies in PubMed, Web of Science and ScienceDirect databases that were published from January 2011 through October 2021. The search string was “rice” AND (“heavy metal” OR “lead” OR “cadmium”) AND “China”. Only studies published in English were considered. Reference lists in selected articles and relevant review articles were manually searched to identify additional studies.

Inclusion and exclusion criteria

After the initial screening, the full text of potentially eligible articles were downloaded and evaluated carefully according to the inclusion and exclusion criteria. The studies were included if they measured levels of Pb and Cd in rice in China, were published in English, and were available as full text. Studies were excluded if they measured metal levels in cooked rice, rice planted on an experimental farm, rice paddies located near mining and smelting areas, or rice samples collected from markets.

Definitions and data extraction

Two authors (M.T.T. and L.S.S.) independently evaluated and extracted data from the included studies using a predefined, standardized protocol. The extracted data on general characteristics of studies included the first author, year of publication, years of sampling, journal of publication, sample size, study area, assay method, average concentration and standard deviation (SD). One study (28) reported ranges, which we converted to SD as described (29). Disagreements about extracted data were resolved through discussion.

Quality assessment

Two authors (X.L.H. and Y.L.W.) independently evaluated the quality of included studies using the Combie evaluation tool (30) based on seven items. Studies scoring 0–4 points were defined as low quality; those scoring 4.0–5.5 points, medium quality; and

those scoring 6.0–7.0 points, high quality. Differences were resolved through discussion.

Statistical analysis and meta-analysis

Meta-analysis was performed using STATA 15.0 software (Stata Corp, College Station, TX, USA). Pooled concentrations and 95% confidence intervals (CIs) were calculated for all outcomes. Statistical heterogeneity among studies was assessed based on I^2, with 25% defined as low heterogeneity; 50%, moderate heterogeneity; and 75%, high heterogeneity (31, 32). Meta-analysis was performed using a random-effects model if $I^2 > 50\%$ (33); otherwise, a fixed-effect model was used. Meta-regression was used to identify studies that might explain the observed heterogeneity; the covariates in this regression were years of sampling, study area, assay method, sample size, and quality score. Sources of heterogeneity were also explored through meta-analysis of subgroups defined by years of sampling, study area, assay method, sample size and quality score.

Sensitivity analysis was conducted by omitting studies one by one, and the P values of pooled concentrations were compared. The results were considered robust if the P values were not substantially different. Publication bias was quantitatively analyzed using Egger's test (34), and risk of bias was considered significant if $P < 0.05$.

Health risk assessment

The target hazard quotient (THQ) developed by the US Environmental Protection Agency (35) was used to assess the potential human health risks associated with long-term exposure to heavy metal pollutants in rice. The THQ was calculated as

$$THQ=\frac{E_F \times E_D \times F_{IR} \times C}{R_{fD} \times W_{AB} \times T_A} \quad , \qquad (1)$$

where E_F is the exposure frequency per year (365 days); E_D, the exposure duration (70 years); F_{IR}, the average daily rice intake in kg person^{-1} day^{-1} (0.389 for adults, 0.198 for children) (27, 36); C, the heavy metal content in rice in mg kg^{-1}; R_{fD}, the oral reference dose for heavy metals in mg kg^{-1} day^{-1} recommended by the US Environmental Protection Agency (0.001 for Cd, 0.0035 for Pb) (35); W_{AB}, the mean body weight in China in kg person^{-1} (55.9 for adults, 32.7 for children) (27, 36); and T_A, the average exposure time (365 days year^{-1} × 70 years).

Total THQ was calculated as

$$TTHQ=\sum THQ \quad , \qquad (2)$$

across all heavy metal pollutants, which in this study were Pb and Cd. THQ / TTHQ < 1 indicated that the food was safe for human consumption (35).

Results and discussion

Study selection

A total of 2130 articles were retrieved from PubMed, Web of Science, and

ScienceDirect databases, and 1561 duplicate articles were excluded. After screening titles and abstracts, we excluded another 327 articles. After carefully reading the full text of the remaining 242 articles, 212 were excluded. Finally, 30 articles were included in the analysis (Fig. 1).

Study characteristics

The main characteristics of the 30 studies are presented in Table 1. The studies were published from January 2011 to October 2021, and they involved a total of 6390 rice samples collected from several major rice-producing areas in China. Among the 30 studies, 24 measured Pb in a total of 5440 rice samples, while 29 studies measured Cd in a total of 6359 rice samples. Concentrations of Pb were determined by inductively coupled plasma-mass spectrometry (ICP-MS, 10 studies), inductively coupled plasma optical emission spectrometry (ICP-OES, 3 studies), atomic absorption spectrometry (AAS, 11 studies), and Cd were determined by inductively coupled plasma-mass spectrometry (15 studies), atomic absorption spectrometry (14 studies).

Assessment of study quality

All studies in the review were judged to be of high or medium quality according to the Combie evaluation tool. The average score was 6.2 points, with 75.9% of the included studies scoring greater than 5.5 points (Table 1).

Meta-analysis of concentrations of Pb and Cd

Of the 30 studies, four were excluded for the meta-analysis of Pb because concentrations were below the limit of detection in three studies (6, 37, 38), while the SD of concentrations in a fourth study (39) was 0.000. In the remaining studies, the pooled concentration of Pb (mg/kg) across several major rice-producing areas in China was 0.10 (95% CI 0.08-0.11; $I^2 = 99.9\%$, $P < 0.001$; Fig. 2). The pooled concentration of Cd (mg/kg) was 0.16 (95% CI 0.14-0.18; $I^2 = 99.4\%$, $P < 0.001$; Fig. 3).

Although some individual studies in our review reported levels of Pb or Cd in rice that exceeded the standard limit in China (0.2 mg/kg), the meta-analysis of pooled data demonstrated that the level of each metal was below this limit.

Publication bias and sensitivity analysis

Egger's test suggested no significant risk of publication bias among studies measuring Pb ($P = 0.712$, Fig. 4A), whereas it suggested significant risk among studies measuring Cd ($P = 0.005$, Fig.4B).

Sensitivity analysis was performed by repeating the meta-analysis after omitting each study one by one and examining whether the results changed substantially. Deletion of each one of the studies did not substantially alter the pooled concentrations of Pb or Cd (Fig. S1 and S2).

Meta-regression analysis

Both uni- and multivariate meta-regressions were conducted with the following covariates: years of sampling, area, assay method, sample size and quality score. Univariate meta-regression for Pb showed that years of sampling, area, assay method, sample size and quality score did not affect outcomes (Table 2). Nevertheless, assay method could explain 16.03% of heterogeneity (adjusted R^2 = 16.03 %, P = 0.046). None of the factors tested substantially affected multivariate meta-regression (Table 3).

Univariate meta-regression for Cd identified the following characteristics as affecting outcomes: northeast vs central China (adjusted R^2 = 47.81%, P = 0.040), eastern vs central China (adjusted R^2 = 47.81%, P＜0.001), southern vs central China (adjusted R^2 = 47.81%, P = 0.007), central vs non-central China (adjusted R^2 = 43.90%, P＜0.001), and sample size (adjusted R^2 = 15.56%, P = 0.016; Table 4). In contrast, years of sampling, assay method and quality score did not affect outcomes. Multivariate meta-regression showed that years of sampling, central vs non-central China, assay method, sample size and quality score were able to explain 41.86% of heterogeneity (Table 5). The P value for the difference between central and non-central China was 0.002.

Meta-analysis showed high heterogeneity for Pb (99.9%) and Cd (99.4%). Uni- and multivariate meta-regression associated the high heterogeneity for Cd to different study

areas in China.

Subgroup analysis

Meta-analysis was repeated for specific subgroups defined in terms of years of sampling, area, assay method, sample size and quality score. Pooled concentrations of Pb (mg/kg) were as follows for different years of sampling (Table 6, Fig. 5A): 2009-2011, 0.10 (95%CI 0.10, 0.10); 2012-2013, 0.07 (95%CI 0.05, 0.10); 2014-2015, 0.07 (95%CI 0.05, 0.08); 2016, 0.19 (95%CI 0.18, 0.20); 2017, 0.09 (95%CI -0.01, 0.19); and 2018, 0.11 (95%CI 0.03, 0.18).

Pooled concentrations of Cd (mg/kg) were as follows for different years of sampling (Table 7, Fig. 5B): 2006, 0.07 (95%CI 0.05, 0.09); 2009-2011, 0.09 (95%CI 0.06, 0.11); 2012-2013, 0.19 (95%CI 0.11, 0.28); 2014-2015, 0.18 (95%CI 0.15, 0.20); 2016, 0.47 (95%CI 0.06, 0.89); 2017, 0.18 (95%CI 0.00, 0.37); 2018, 0.11 (95%CI 0.06, 0.16).

Regardless of years of sampling, levels of Pb were below the limit defined by China as safe. In contrast, the level of Cd exceeded the standard limit in 2016, but not in other years.

Pooled concentrations of Pb (mg/kg) were 0.26 (95%CI 0.25, 0.27) for northeast China, but 0.10 (95%CI 0.08, 0.12) across all other regions (Table 6, Fig. 6A). Pooled

concentrations of Cd (kg/mg) were 0.43 (95%CI 0.27, 0.60) in central China, followed by 0.21 (95%CI 0.15, 0.27) in southern China, below 0.20 in other areas and 0.13 (95%CI 0.11, 0.15) across all non-central regions (Fig. 6B). Heterogeneity was high for Cd measurements in central China (I^2 = 96.4%) as well as non-central regions (99.5%; Table 7).

Pooled concentrations of Pb (mg/kg) were as follows for different assay methods: ICP-MS, 0.06 (95%CI 0.04, 0.09); ICP-OES, 0.13 (95%CI 0.01, 0.25); and AAS, 0.15 (95%CI 0.08, 0.22) (Table 6). Pooled concentrations of Cd (mg/kg) were 0.16 (95%CI 0.14, 0.18) for ICP-MS and 0.16 (95%CI 0.13, 0.20) for AAS (Table 7).

Pooled concentrations of Pb (mg/kg) were 0.10 (95%CI 0.07, 0.13) among small studies (≤150 samples) and 0.09 (95%CI 0.07, 0.10) among large studies (>150 samples) (Table 6). Pooled concentrations of Cd (mg/kg) were 0.12 (95%CI 0.10, 0.14) among small studies and 0.27 (95%CI 0.21, 0.33) among large studies (Table 7).

Among studies measuring Pb, 18 were assigned to high quality and gave a pooled concentration of 0.10 (95%CI 0.08, 0.11) mg/kg. Four studies were assigned to medium quality and gave a pooled concentration of 0.13 (95%CI -0.05, 0.30) mg/kg (Table 6). Among studies measuring Cd, 24 were assigned to high quality and gave a pooled concentration of 0.19 (95%CI 0.17, 0.21) mg/kg. Nine studies were assigned to medium

quality and gave a pooled concentration of 0.09 (95%CI 0.05, 0.13) mg/kg (Table 7).

Our meta-analysis indicated more serious contamination of rice with Cd than with Pb. Contamination with Cd appears particularly severe in the central region of China (0.43 mg/kg), based primarily on pooled data from Hunan (11, 40-44) but also some data from Jiangxi and Hubei (13). Our findings are consistent with several studies reporting widespread soil contamination with Cd in Hunan, where some types of local rice are referred to as "cadmium rice" (11, 45, 46).

Although our studies sampled from all six of the major rice-producing regions in China, the sampling was concentrated in Zhejiang in the Yangtze River Delta and Guangdong in southern China. Given that levels of heavy metals in rice appear to vary geographically (23), we recommend that future studies focus on neglected rice-producing regions in China in order to provide a more comprehensive and accurate picture of heavy metal contamination.

Health risk assessment

Our meta-analysis of the literature suggests a Pb THQ of 0.20 for adults and 0.17 for children (Table 8), both of which are below 1.0, indicating safe levels in rice. In contrast, the Cd THQ was 1.11 for adults and 0.97 for children, indicating a health concern for adults but not children. Combining the THQs for Pb and Cd led to a total THQ higher

than 1 for adults and children. This suggests a serious health risk for children and adults.

Conclusions

Our meta-analysis suggests that pooled Pb and Cd levels are within the limits specified by Chinese food safety standards. Nevertheless, the total target hazard quotient for both metals appears to exceed 1.0 for adults and children, suggesting that rice consumption poses a health risk and more should be done to control heavy metal pollution of soils in rice paddies in China.

Funding

This work was supported by the National Key R&D Program of China (2017YFC1602000), and the Chongqing Science and Technology Bureau (cstc2021jxjl130009) and the Key demonstration project of Chongqing Technology Innovation and application demonstration project (cstc2018jscx-mszdX0122).

Disclosure statement

No potential conflict of interest was reported by the author(s).

Author Contributions

Conceptualization, X.S.Y; Data curation, M.T.T and L.S.S; Formal analysis, X.L.H and M.T.T; Funding acquisition, X.S.Y and X.L.H; Investigation, X.L.H; Project administration, H.Y.Z; Resources, Y.L.W and G.R.F; Supervision, B.Z, S.Q.C and

H.Y.Z; Writing – original draft, X.L.H, B.Z and Y.L.W.

References

1. Ren Y, Lin M, Liu Q, Zhang Z, Fei X, Xiao R, et al. Contamination assessment, health risk evaluation, and source identification of heavy metals in the soil-rice system of typical agricultural regions on the southeast coast of China. Environmental Science and Pollution Research. 2021;28(10):12870-80.

2. Cui ZW, Wang Y, Zhao N, Yu R, Xu GH, Yu Y. Spatial distribution and risk assessment of heavy metals in paddy soils of Yongshuyu irrigation area from Songhua river basin, Northeast China. Chin Geogr Sci. 2018;28(5):797-809.

3. Tang L, Deng SH, Tan D, Long JM, Lei M. Heavy metal distribution, translocation, and human health risk assessment in the soil-rice system around Dongting Lake area, China. Environmental Science and Pollution Research. 2019;26(17):17655-65.

4. Rogan N, Serafimovski T, Dolenec M, Tasev G, Dolenec T. Heavy metal contamination of paddy soils and rice (Oryza sativa L.) from Koani Field (Macedonia). Environ Geochem Health. 2009;31(4):439-51.

5. Romkens P, Guo HY, Chu CL, Liu TS, Chiang CF, Koopmans GF. Prediction of Cadmium uptake by brown rice and derivation of soil-plant transfer models to improve soil protection guidelines. Environmental Pollution. 2009;157(8-9):2435-44.

6. Gao Z, Fu W, Zhang M, Zhao K, Tunney H, Guan Y. Potentially hazardous metals contamination in soil-rice system and it's spatial variation in Shengzhou City, China. Journal of Geochemical Exploration. 2016;167:62-9.

7. NHCPRC. National Health Commission of the People’s Republic of China. China

National Food Safety Standard Maximum Limit of Contaminants in Food (GB 2762-2017) 2017.

8. Zhai WW, Zhao WL, Yuan HH, Guo T, Hashmi MZ, Liu XM, et al. Reduced Cd, Pb, and As accumulation in rice (Oryza sativa L.) by a combined amendment of calcium sulfate and ferric oxide. Environmental Science and Pollution Research. 2020;27(2):1348-58.

9. Cai LM, Wang QS, Luo J, Chen LG, Zhu RL, Wang S, et al. Heavy metal contamination and health risk assessment for children near a large Cu-smelter in central China. Science of the Total Environment. 2019;650:725-33.

10. Zhuang P, Sun S, Li YW, Li F, Zou B, Li YX, et al. Oral bioaccessibility and exposure risk of metal(loid)s in local residents near a mining-impacted area, Hunan, China. International Journal of Environmental Research and Public Health. 2018;15(8):11.

11. Chen H, Yang X, Wang P, Wang Z, Li M, Zhao F-J. Dietary cadmium intake from rice and vegetables and potential health risk: A case study in Xiangtan, southern China. Science of the Total Environment. 2018;639:271-7.

12. Wang Q, Hao D, Wang F, Wang H, Huang X, Li F, et al. Development of a new framework to estimate the environmental risk of heavy metal(loid)s focusing on the spatial heterogeneity of the industrial layout. Environment International. 2021;147.

13. Liu Z, Zhang Q, Han T, Ding Y, Sun J, Wang F, et al. Heavy metal pollution in a soil-rice system in the Yangtze River region of China. International Journal of

Environmental Research and Public Health. 2016;13(1).

14. Huang Z, Pan XD, Wu PG, Han JL, Chen Q. Health risk assessment of heavy metals in rice to the population in Zhejiang, China. PLoS One. 2013;8(9):6.

15. Karalliedde L, Brooke N. Essentials of Toxicology for Health Protection. Toxicity of heavy metals and trace elements: Oxford University Press; 2013.

16. Liu KL, Zheng JB, Chen FS. Effects of washing, soaking and domestic cooking on cadmium, arsenic and lead bioaccessibilities in rice. J Sci Food Agric. 2018;98(10):3829-35.

17. Godt J, Scheidig F, Grosse-Siestrup C, Esche V, Brandenburg P, Reich A, et al. The toxicity of cadmium and resulting hazards for human health. J Occup Med Toxicol. 2006;1:6.

18. Kim KW, Thornton I. Influence of uraniferous black shales on cadmium, molybdenum and selenium in soils and crop plants in the deog-pyoung area of Korea. Environ Geochem Health. 1993;15(2-3):119-33.

19. ATSDR. Agency for Toxic Substances and Disease Registry. Toxicological profile for cadmium. 2012.

20. Flora G, Gupta D, Tiwari A. Toxicity of lead: A review with recent updates. Interdisciplinary toxicology. 2012;5(2):47-58.

21. Ashraf U, Kanu AS, Mo ZW, Hussain S, Anjum SA, Khan I, et al. Lead toxicity in rice: effects, mechanisms, and mitigation strategies-a mini review. Environmental Science and Pollution Research. 2015;22(23):18318-32.

22. Norton GJ, Williams PN, Adomako EE, Price AH, Zhu YG, Zhao FJ, et al. Lead in rice: Analysis of baseline lead levels in market and field collected rice grains. Science of the Total Environment. 2014;485:428-34.

23. Fang Y, Sun X, Yang W, Ma N, Xin Z, Fu J, et al. Concentrations and health risks of lead, cadmium, arsenic, and mercury in rice and edible mushrooms in China. Food Chemistry. 2014;147:147-51.

24. Xie LH, Tang SQ, Wei XJ, Shao GN, Jiao GA, Sheng ZH, et al. The cadmium and lead content of the grain produced by leading Chinese rice cultivars. Food Chemistry. 2017;217:217-24.

25. Mu T, Wu T, Zhou T, Li Z, Ouyang Y, Jiang J, et al. Geographical variation in arsenic, cadmium, and lead of soils and rice in the major rice producing regions of China. Science of the Total Environment. 2019;677:373-81.

26. Kong X, Liu T, Yu Z, Chen Z, Lei D, Wang Z, et al. Heavy Metal Bioaccumulation in Rice from a High Geological Background Area in Guizhou Province, China. International Journal of Environmental Research and Public Health. 2018;15(10).

27. Zheng N, Wang Q, Zhang X, Zheng D, Zhang Z, Zhang S. Population health risk due to dietary intake of heavy metals in the industrial area of Huludao city, China. Science of the Total Environment. 2007;387(1-3):96-104.

28. Lian M, Wang J, Sun L, Xu Z, Tang J, Yan J, et al. Profiles and potential health risks of heavy metals in soil and crops from the watershed of Xi River in Northeast China. Ecotoxicology and Environmental Safety. 2019;169:442-8.

29. Hozo SP, Djulbegovic B, Hozo I. Estimating the mean and variance from the median, range, and the size of a sample. BMC medical research methodology. 2005;5:13.

30. Si YH, Wang CY, Guo Y, Yin H, Ma Y. Prevalence of osteoporosis in patients with type 2 diabetes mellitus in the Chinese mainland A protocol of systematic review and meta-analysis. Medicine. 2020;99(16):5.

31. Zhou ZK, Liang YF, Zhang XQ, Xu JJ, Lin JY, Zhang RW, et al. Low-density Lipoprotein Cholesterol and Alzheimer's disease: A systematic review and meta-analysis. Front Aging Neurosci. 2020;12:11.

32. Higgins JPT, Thompson SG, Deeks JJ, Altman DG. Measuring inconsistency in meta-analyses. Br Med J. 2003;327(7414):557-60.

33. Higgins JPT, Thompson SG. Quantifying heterogeneity in a meta-analysis. Stat Med. 2002;21(11):1539-58.

34. Egger M, Smith GD, Schneider M, Minder C. Bias in meta-analysis detected by a simple, graphical test. BMJ-British Medical Journal. 1997;315(7109):629-34.

35. EPA U. Risk-based concentration table. United States Environmental Protection Agency Washington DC, Philadelphia. 2000.

36. Khan S, Cao Q, Zheng YM, Huang YZ, Zhu YG. Health risks of heavy metals in contaminated soils and food crops irrigated with wastewater in Beijing, China. Environmental Pollution. 2008;152(3):686-92.

37. He M, Shen H, Li Z, Wang L, Wang F, Zhao K, et al. Ten-year regional monitoring

of soil-rice grain contamination by heavy metals with implications for target remediation and food safety. Environmental Pollution. 2019;244:431-9.

38. Zhao K, Fu W, Ye Z, Zhang C. Contamination and spatial variation of heavy metals in the soil-rice system in Nanxun county, Southeastern China. International Journal of Environmental Research and Public Health. 2015;12(2):1577-94.

39. Li Z, Su H, Wang L, Hu D, Zhang L, Fang J, et al. Epidemiological Study on Metal Pollution of Ningbo in China. International Journal of Environmental Research and Public Health. 2018;15(3).

40. Mao C, Song Y, Chen L, Ji J, Li J, Yuan X, et al. Human health risks of heavy metals in paddy rice based on transfer characteristics of heavy metals from soil to rice. Catena. 2019;175:339-48.

41. Lu A, Li B, Li J, Chen W, Xu L. Heavy metals in paddy soil-rice systems of industrial and township areas from subtropical China: Levels, transfer and health risks. Journal of Geochemical Exploration. 2018;194:210-7.

42. Zeng F, Wei W, Li M, Huang R, Yang F, Duan Y. Heavy metal contamination in rice-producing soils of Hunan province, China and potential health risks. International Journal of Environmental Research and Public Health. 2015;12(12):15584-93.

43. Zhang Z, Zhang N, Li H, Lu Y, Yang Z. Potential health risk assessment for inhabitants posed by heavy metals in rice in Zijiang River basin, Hunan Province, China. Environmental Science and Pollution Research. 2020;27(19):24013-24.

44. Du F, Yang Z, Liu P, Wang L. Accumulation, translocation, and assessment of

heavy metals in the soil-rice systems near a mine-impacted region. Environmental Science and Pollution Research. 2018;25(32):32221-30.

45. Zhao F-J, Ma Y, Zhu Y-G, Tang Z, McGrath SP. Soil contamination in China: Current status and mtigation strategies. Environmental Science & Technology. 2015;49(2):750-9.

46. Zhu H, Chen C, Xu C, Zhu Q, Huang D. Effects of soil acidification and liming on the phytoavailability of cadmium in paddy soils of central subtropical China. Environmental Pollution. 2016;219:99-106.

Table 1. Main characteristics of studies included in the meta-analysis

No.	Study	Year(s) of sampling	Area	Sample size	Level (mg/kg dry weight), mean±SD		Assay method	Quality (Combie points)
					Pb	Cd		
1	Zhao et al., 2011	2006	Zhejiang (Wenling)	96	NR	0.072±0.105	GFAAS	Medium (5.5)
2	Hu et al., 2013	2009-2011	Northeast/Northern China/Northwest/Eastern China/Central China/Southern China/Southwest	92	0.10±0.14	0.08±0.07	GFAAS	High (6.5)
3	Li et al., 2014	2011	Zhejiang (Wenling)	219	NR	0.132±0.24	GFAAS	High (6.5)
4	Mao et al., 2019	2011	Yangtze River Delta (Jiangsu, Zhejiang, Shanghai)	137	0.098±0.003	0.064±0.008	ICP-MS	High (6.5)
5	Liu et al., 2016	2012	Yangtze River Region (Hubei, Hunan, Jiangxi)	101	0.25±0.11	0.29±0.39	GFAAS	High (6.0)
6	Xie et al., 2017	2012-2013	18 provinces	110	0.0435±0.0755	0.0650±0.1266	GFAAS	High (6.5)
7	Gao et al., 2016	2013	Zhejiang (Shengzhou)	94	UD	0.09±0.10	GFAAS	High (6.5)
8	Hu et al., 2019	2013	South of Yangtze River Delta (Zhejiang)	915	0.060±0.08	0.08±0.07	Pb: ICP-OES<br>Cd: ICP-MS	High (6.5)

No.	Study	Year(s) of sampling	Area	Sample size	Level (mg/kg dry weight), mean±SD		Assay method	Quality (Combie points)
					Pb	Cd		
9	Lu et al., 2018	2013	Hunan	440	0.049±0.004	0.565±0.376	AAS	High (6.0)
10	Li et al., 2018	2013	Yangtze River Delta region (Ningbo)	Rural: 10<br>Industrial: 10	0.027±0.034<br>0.004±0.000	0.071±0.061<br>0.132±0.043	ICP-MS	Medium (5.5)
11	Zeng et al., 2015	2013	Hunan	28	0.022±0.021	0.312±0.434	GFAAS	High (6.0)
12	Tang et al., 2021	2014	Guangxi (Liujiang District, Southern part of Liuzhou)	75	NR	0.16±0.22	ICP-MS	High (6.5)
13	Zheng et al., 2020	2014	Pearl River Delta	879	0.27±0.59	0.17±0.20	Pb: FAAS<br>Cd: GFAAS	High (6.5)
14	Huang et al., 2018	2014-2015	Southeast China (Zhejiang)	32	0.18±0.08	0.21±0.07	Pb: ICP-OES<br>Cd: ICP-MS	High (6.5)
15	Gu et al., 2019	2015	Guangxi (Nanning and Laibin)	246	0.042±0.020	0.182±0.171	ICP-MS	High (6.5)
16	Mu et al., 2019	2015	19 provinces<br>South/ Yangtze River Delta /West	113<br>574	0.036±0.021<br>0.036±0.017	0.087±0.174<br>0.199±0.406	ICP-MS	High (6.5)
17	Ma et al., 2017	2015	Guangdong	48	0.0274±0.0202	0.231±0.222	ICP-MS	High (6.0)
18	Chen et al., 2018	2016	Hunan (Xiangtan)	200	NR	0.69±0.60	ICP-MS	High (6.5)

No.	Study	Year(s) of sampling	Area	Sample size	Level (mg/kg dry weight), mean±SD		Assay method	Quality (Combie points)
					Pb	Cd		
19	He et al., 2019	2016	Zhejiang (Wenling)	169	UD	0.117±0.189	GFAAS	High (6.5)
20	Wang et al., 2021	2016	Guangdong (Shaoguan)	570	0.19±0.092	0.62±0.94	Pb: FAAS<br>Cd: GFAAS	High (6.5)
21	Ren et al., 2021	2017	Northern part of Zhejiang province	120	0.04 ±0.05	0.09±0.07	ICP-MS	High (6.0)
22	Zhang et al., 2020	2017	Central part of Hunan	135	0.145±0.328	0.283±0.330	ICP-MS	High (6.5)
23	Guo et al., 2020	2018	Centre of Zhejiang (Jin-Qu Basin)	86	0.148±0.094	0.163±0.206	ICP-MS	High (7.0)
24	Liu et al., 2020	2018	Pearl River Delta (Zhuhai)	70	NR	0.12±0.08	ICP-MS	High (6.0)
25	Lu et al., 2021	2018	Southwest of Fujian (Longyang)	332	0.072±0.085	0.064±0.075	ICP-MS	High (7.0)
26	Du et al., 2018	NR	Hunan (Southern part of Changsha)	27	0.031±0.023	0.291±0.295	ICP-MS	Medium (5.0)
27	Lian et al., 2019	NR	Shenyang	41	0.26±0.026	0.14±0.016	GFAAS	Medium (5.5)
28	Yu et al., 2019	NR	Zhejiang (Nanxun，Shengzhou，Wenling)	Nanxun: 100<br>Shengzhou: 94<br>Wenling: 96	NR<br>NR<br>NR	0.011±0.015<br>0.09±0.10<br>0.072±0.105	GFAAS	Medium (5.0)

No.	Study	Year(s) of sampling	Area	Sample size	Level (mg/kg dry weight), mean±SD		Assay method	Quality (Combie points)
					Pb	Cd		
29	Zhang et al., 2018	NR	Guangdong (Sihui)	31	2.05±4.67	NR	ICP-OES	Medium (5.5)
30	Zhao et al., 2015	NR	Zhejiang (Nanxun)	100	UD	0.011±0.015	GFAAS	Medium (5.5)

AAS, atomic absorption spectrometry; FAAS, flame atomic absorption spectrometry; GFAAS, graphite furnace atomic absorption spectrometry; ICP, inductively coupled plasma; MS, mass spectrometry; NR, not reported; OES, optical emission spectroscopy; UD, undetectable (below the detection limit).

Table 2. Univariate meta-regression for Pb

Covariate	Coefficient	95% confidence interval	Adjusted R^2	P
**Years of sampling**	0.0065976	-0.0196145 to 0.0328096	-4.36%	0.602
**Area of China**				
E vs N	-0.1681435	-0.3993931 to 0.0631061	2.31%	0.141
C vs N	-0.161892	-0.3967119 to 0.0729279	2.31%	0.161
S vs N	-0.1370138	-0.3715523 to 0.0975248	2.31%	0.231
N vs non-N	0.1567184	-0.045606 to -0.045606	14.13%	0.120
**Assay method**				
ICP-MS vs AAS	-0.0842295	-0.1668027 to -0.0016563	16.03%	0.046
ICP-OES vs AAS	-0.0201361	-0.1604507 to 0.1201785	16.03%	0.767
**Sample size**	0.0000177	-0.091281 to 0.0913164	-5.42%	1.000
**Quality score**	-0.0134979	-0.1359797 to 0.1089838	-5.34%	0.821

Regions of China were classified as follows: E, eastern (Zhejiang, Jiangsu, Shanghai); N, northeast (Liaoning); C, central (Hubei, Hunan, Jiangxi); S, southern (Guangxi, Guangdong, Fujian).

AAS, atomic absorption spectrometry; ICP, inductively coupled plasma; MS, mass spectrometry; OES, optical emission spectrometry.

Table 3. Multivariate meta-regression for Pb

Covariate	Coefficient	95% confidence interval	Adjusted R^2	P
**Years of sampling**	0.0186612	-0.0203312 to 0.0576536		0.307
**Assay method**				
ICP-MS vs AAS	-0.1118847	-0.2457248 to 0.0219555	-3.89%	0.091
ICP-OES vs AAS	-0.036351	-0.1967322 to 0.1240303		0.620
**Sample size**	-0.0305863	-0.1400625 to 0.0788899		0.543
**Quality score**	0.0229515	-0.198218 to 0.2441209		0.820

Assay methods are defined in Table 2.

Table 4. Univariate meta-regression for Cd

Covariate	Coefficient	95% confidence interval	Adjusted R^2	P
**Years of sampling**	0.0152284	-0.0275551 to 0.0580119	-2.00%	0.470
**Area of China**				
N vs C	-0.2968999	-0.5788897 to -0.0149101	47.81%	0.040
E vs C	-0.3322005	-0.4702123 to -0.1941887	47.81%	0.000
S vs C	-0.2211105	-0.3775602 to -0.0646608	47.81%	0.007
C vs non-C	0.2980667	0.1612039 to 0.4349295	43.90%	0.000
**Assay method**	-0.0071547	-0.1240696 to 0.1097602	-3.38%	0.901
**Sample size**	0.1437373	0.0285398 to 0.2589348	15.56%	0.016
**Quality score**	0.1109727	-0.0133534 to 0.2352988	7.26%	0.078

Abbreviations for regions of China are defined in Table 2.

Table 5. Multivariate meta-regression for Cd

Covariate	Coefficient	95% confidence interval	Adjusted R^2	P
Years of sampling	0.0092293	-0.0370372 to 0.0554958	41.86%	0.679
Area: central vs non-central	0.2869248	0.1182071 to 0.4556425		0.002
Assay method	0.0520104	-0.0969596 to 0.2009805		0.471
Sample size	0.0768156	-0.0605715 to 0.2142028		0.254
Quality score	0.024864	-0.1877351 to 0.2374632		0.808

Table 6. Subgroup analysis of Pb concentrations in rice.

Stratifying variable	Subgroup	No. of studies	Sample size	Concentration, mg/kg (95%CI)	P	I^2 (%)
Years of sampling	2009-2011	2	229	0.10 (0.10, 0.10)	0.891	0.0
	2012-2013	6	1604	0.07 (0.05, 0.10)	＜0.001	98.8
	2014-2015	6	1892	0.07 (0.05, 0.08)	＜0.001	98.1
	2016	1	570	0.19 (0.18, 0.20)	/	/
	2017	2	255	0.09 (-0.01, 0.19)	＜0.001	92.6
	2018	2	418	0.11 (0.03, 0.18)	＜0.001	97.8
	Not reported	3	99	0.18 (-0.04, 0.40)	＜0.001	99.9
Area of China	Multiple areas	4	889	0.04 (0.03, 0.05)	＜0.001	85.2
	Northeast	1	41	0.26 (0.25, 0.27)	/	/
	Eastern	6	1300	0.09 (0.06, 0.12)	＜0.001	98.9
	Central	5	731	0.09 (0.06, 0.13)	＜0.001	99.0
	Southern	6	2106	0.12 (0.06, 0.18)	＜0.001	99.7
	Northeast	1	41	0.26 (0.25, 0.27)	/	/
	Non-Northeast	17	4137	0.10 (0.08, 0.12)	＜0.001	99.9
Assay method	ICP-MS	11	1828	0.06 (0.04, 0.09)	＜0.001	99.9
	ICP-OES	3	978	0.13 (0.01, 0.25)	＜0.001	97.3
	AAS	8	2261	0.15 (0.08, 0.22)	＜0.001	99.8
Sample size	≤150	15	1111	0.10 (0.07, 0.13)	＜0.001	99.7
	＞150	7	3956	0.09 (0.07, 0.10)	＜0.001	99.7
Quality score	High	18	4958	0.10 (0.08, 0.11)	＜0.001	99.9
	Medium	4	109	0.13 (-0.05, 0.30)	＜0.001	99.8

Areas of China and assay methods are defined in Table 2.

Table 7. Subgroup analysis of Cd concentrations in rice.

Stratifying variable	Subgroup	No. of studies	Sample size	Concentration 95%CI	P	I^2 (%)
Years of sampling	2006	1	96	0.07 (0.05, 0.09)	/	/
	2009-2011	3	448	0.09 (0.06, 0.11)	＜0.001	91.0
	2012-2013	8	1708	0.19 (0.11, 0.28)	＜0.001	99.1
	2014-2015	7	1967	0.18 (0.15, 0.20)	＜0.001	86.1
	2016	3	939	0.47 (0.06, 0.89)	＜0.001	99.3
	2017	2	255	0.18 (-0.00, 0.37)	＜0.001	97.7
	2018	3	488	0.11 (0.06, 0.16)	＜0.001	95.6
	Not reported	6	458	0.09 (0.04, 0.14)	＜0.001	99.8
Area of China	Multiple areas	4	889	0.11 (0.06, 0.15)	＜0.001	93.7
	Northeast	1	41	0.14 (0.14, 0.14)	/	/
	Eastern	16	2379	0.10 (0.08, 0.12)	＜0.001	99.4
	Central	5	830	0.43 (0.27, 0.60)	＜0.001	96.4
	Southern	7	2220	0.21 (0.15, 0.27)	＜0.001	98.6
	Non-Central	24	4640	0.13 (0.11, 0.15)	＜0.001	99.5
Assay method	ICP-MS	17	3130	0.16 (0.14, 0.18)	＜0.001	97.9
	AAS	16	3229	0.16 (0.13, 0.20)	＜0.001	99.6
Sample size	⩽150	23	1815	0.12 (0.10, 0.14)	＜0.001	99.4
	＞150	10	4544	0.27 (0.21, 0.33)	＜0.001	99.4
Quality score	High	24	5785	0.19 (0.17, 0.21)	＜0.001	98.9
	Medium	9	574	0.09 (0.05, 0.13)	＜0.001	99.7

Areas of China and assay methods are defined in Table 2.

Table 8. THQ and total THQ of Pb and Cd due to rice consumption

Group	Pb-THQ	Cd-THQ	Total THQ
Adults	0.20	1.11	1.31
Children	0.17	0.97	1.14

THQ, target hazard quotient.

Figure Click here to access/download;Figure;Figure.docx

Figure captions

Figure 1 Flow diagram of study inclusion in the meta-analysis.

Figure 2 Meta-analysis of Pb concentrations in rice.

Figure 3 Meta-analysis of Cd concentrations in rice.

Figure 4 Egger's test to assess risk of publication bias among studies measuring (A) Pb or (B) Cd in rice samples.

Figure 5 Pooled concentrations of (A) Pb and (B) Cd in different years of sampling. The dashed line indicates the safety limit defined by the Chinese government. dw, dry weight.

Figure 6 Pooled concentrations of (A) Pb and (B) Cd in different areas of China. Regions of China are defined as in Table 2. ✦ indicates exceed the standard limit.

Figure 1

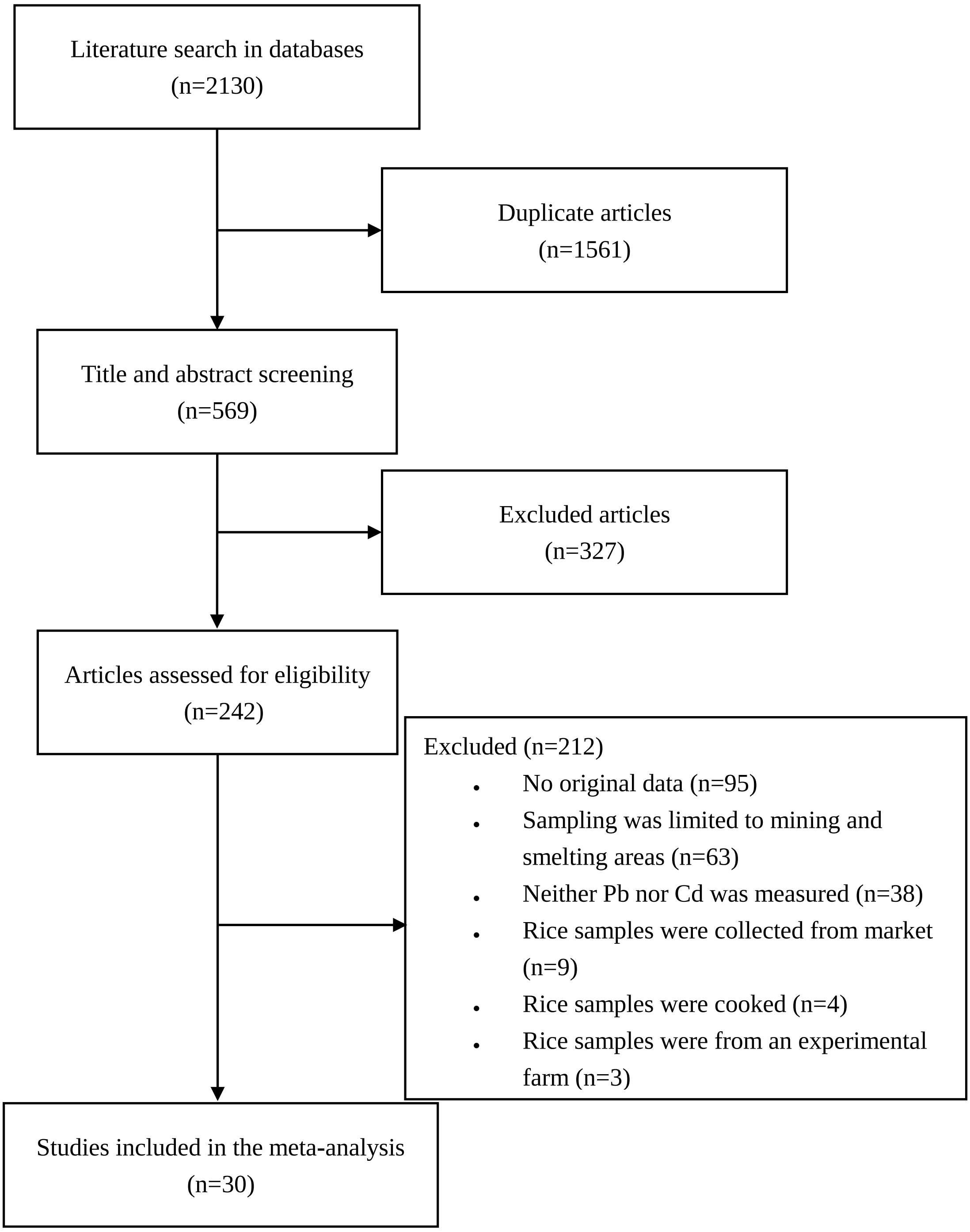
Literature search in databases
(n=2130)
Duplicate articles
(n=1561)
Title and abstract screening
(n=569)
Excluded articles
(n=327)
Articles assessed for eligibility
(n=242)
Excluded (n=212)
• No original data (n=95)
• Sampling was limited to mining and smelting areas (n=63)
• Neither Pb nor Cd was measured (n=38)
• Rice samples were collected from market (n=9)
• Rice samples were cooked (n=4)
• Rice samples were from an experimental farm (n=3)
Studies included in the meta-analysis
(n=30)

Figure 2

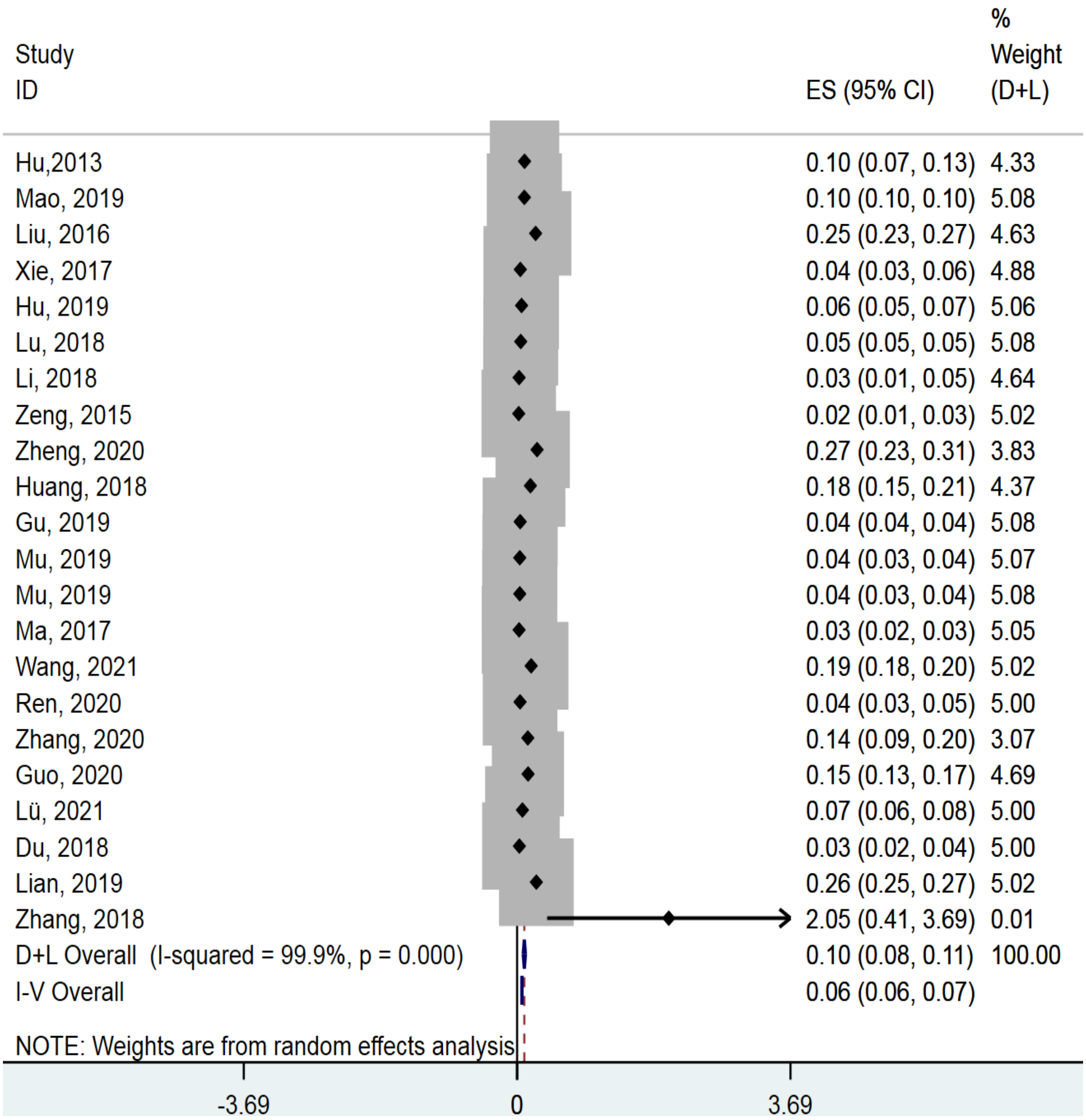
Study ID
ES (95% CI)
% Weight (D+L)
Hu,2013 0.10 (0.07, 0.13) 4.33
Mao, 2019 0.10 (0.10, 0.10) 5.08
Liu, 2016 0.25 (0.23, 0.27) 4.63
Xie, 2017 0.04 (0.03, 0.06) 4.88
Hu, 2019 0.06 (0.05, 0.07) 5.06
Lu, 2018 0.05 (0.05, 0.05) 5.08
Li, 2018 0.03 (0.01, 0.05) 4.64
Zeng, 2015 0.02 (0.01, 0.03) 5.02
Zheng, 2020 0.27 (0.23, 0.31) 3.83
Huang, 2018 0.18 (0.15, 0.21) 4.37
Gu, 2019 0.04 (0.04, 0.04) 5.08
Mu, 2019 0.04 (0.03, 0.04) 5.07
Mu, 2019 0.04 (0.03, 0.04) 5.08
Ma, 2017 0.03 (0.02, 0.03) 5.05
Wang, 2021 0.19 (0.18, 0.20) 5.02
Ren, 2020 0.04 (0.03, 0.05) 5.00
Zhang, 2020 0.14 (0.09, 0.20) 3.07
Guo, 2020 0.15 (0.13, 0.17) 4.69
Lü, 2021 0.07 (0.06, 0.08) 5.00
Du, 2018 0.03 (0.02, 0.04) 5.00
Lian, 2019 0.26 (0.25, 0.27) 5.02
Zhang, 2018 2.05 (0.41, 3.69) 0.01
D+L Overall (I-squared = 99.9%, p = 0.000) 0.10 (0.08, 0.11) 100.00
I-V Overall 0.06 (0.06, 0.07)
NOTE: Weights are from random effects analysis
-3.69
0
3.69

Figure 3

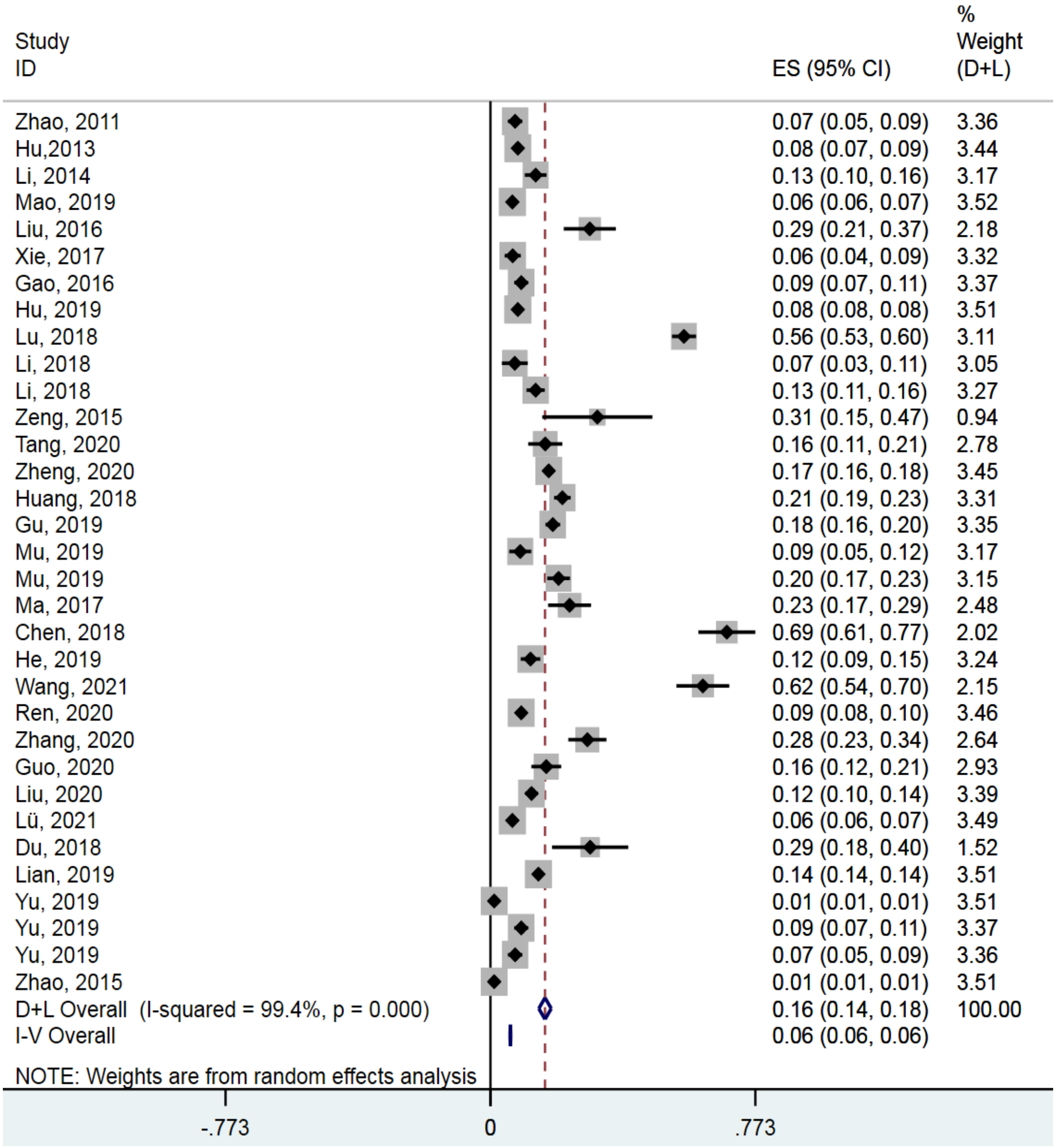
Study
ID
ES (95% CI)
%
Weight
(D+L)
Zhao, 2011 0.07 (0.05, 0.09) 3.36
Hu,2013 0.08 (0.07, 0.09) 3.44
Li, 2014 0.13 (0.10, 0.16) 3.17
Mao, 2019 0.06 (0.06, 0.07) 3.52
Liu, 2016 0.29 (0.21, 0.37) 2.18
Xie, 2017 0.06 (0.04, 0.09) 3.32
Gao, 2016 0.09 (0.07, 0.11) 3.37
Hu, 2019 0.08 (0.08, 0.08) 3.51
Lu, 2018 0.56 (0.53, 0.60) 3.11
Li, 2018 0.07 (0.03, 0.11) 3.05
Li, 2018 0.13 (0.11, 0.16) 3.27
Zeng, 2015 0.31 (0.15, 0.47) 0.94
Tang, 2020 0.16 (0.11, 0.21) 2.78
Zheng, 2020 0.17 (0.16, 0.18) 3.45
Huang, 2018 0.21 (0.19, 0.23) 3.31
Gu, 2019 0.18 (0.16, 0.20) 3.35
Mu, 2019 0.09 (0.05, 0.12) 3.17
Mu, 2019 0.20 (0.17, 0.23) 3.15
Ma, 2017 0.23 (0.17, 0.29) 2.48
Chen, 2018 0.69 (0.61, 0.77) 2.02
He, 2019 0.12 (0.09, 0.15) 3.24
Wang, 2021 0.62 (0.54, 0.70) 2.15
Ren, 2020 0.09 (0.08, 0.10) 3.46
Zhang, 2020 0.28 (0.23, 0.34) 2.64
Guo, 2020 0.16 (0.12, 0.21) 2.93
Liu, 2020 0.12 (0.10, 0.14) 3.39
Lü, 2021 0.06 (0.06, 0.07) 3.49
Du, 2018 0.29 (0.18, 0.40) 1.52
Lian, 2019 0.14 (0.14, 0.14) 3.51
Yu, 2019 0.01 (0.01, 0.01) 3.51
Yu, 2019 0.09 (0.07, 0.11) 3.37
Yu, 2019 0.07 (0.05, 0.09) 3.36
Zhao, 2015 0.01 (0.01, 0.01) 3.51
D+L Overall (I-squared = 99.4%, p = 0.000) 0.16 (0.14, 0.18) 100.00
I-V Overall 0.06 (0.06, 0.06)
NOTE: Weights are from random effects analysis
-.773
0
.773

Figure 4

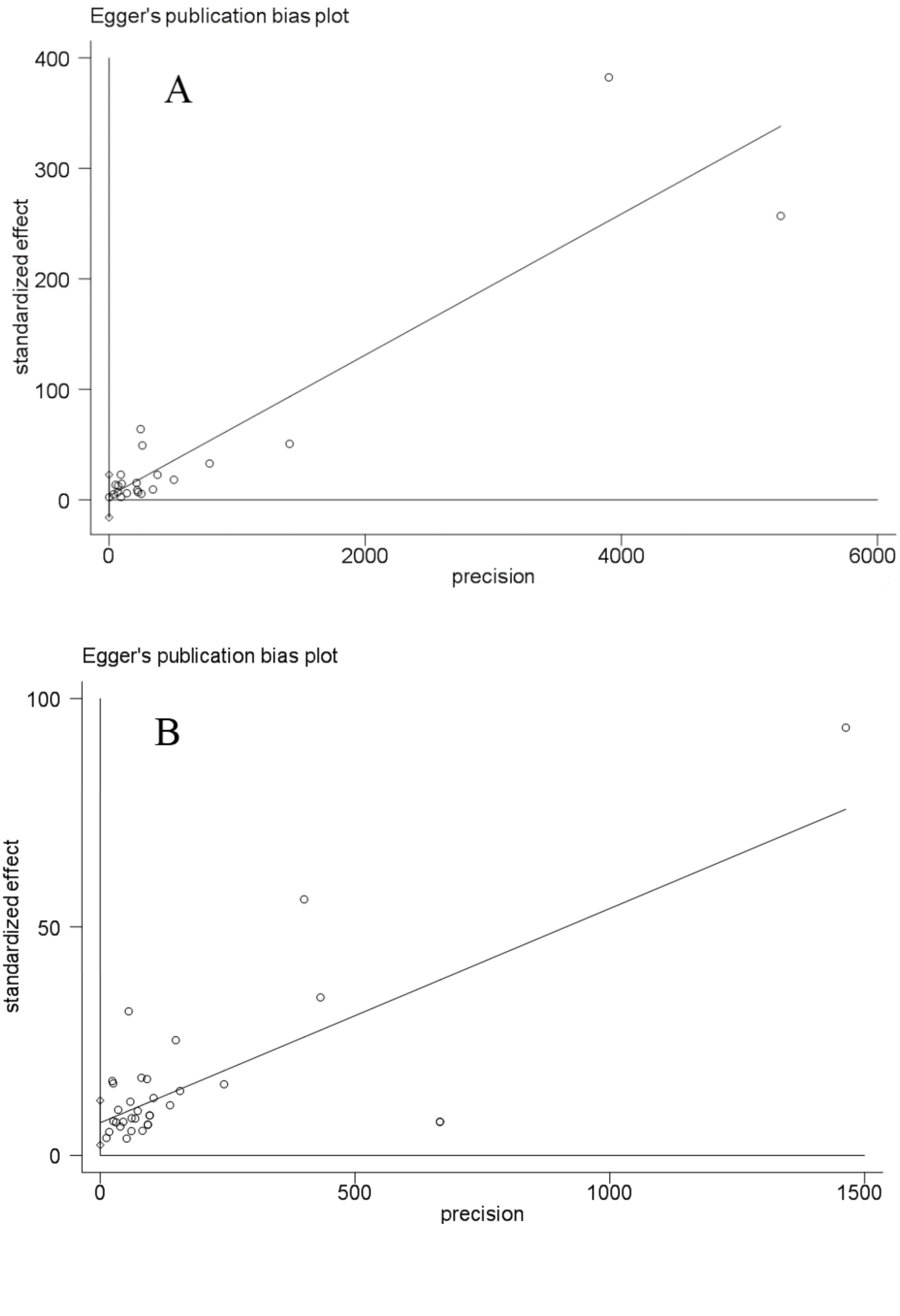

Egger's publication bias plot
A
400
300
200
100
0
standardized effect
0
2000
4000
6000
precision
Egger's publication bias plot
B
100
50
0
standardized effect
0
500
1000
1500
precision

Figure 5

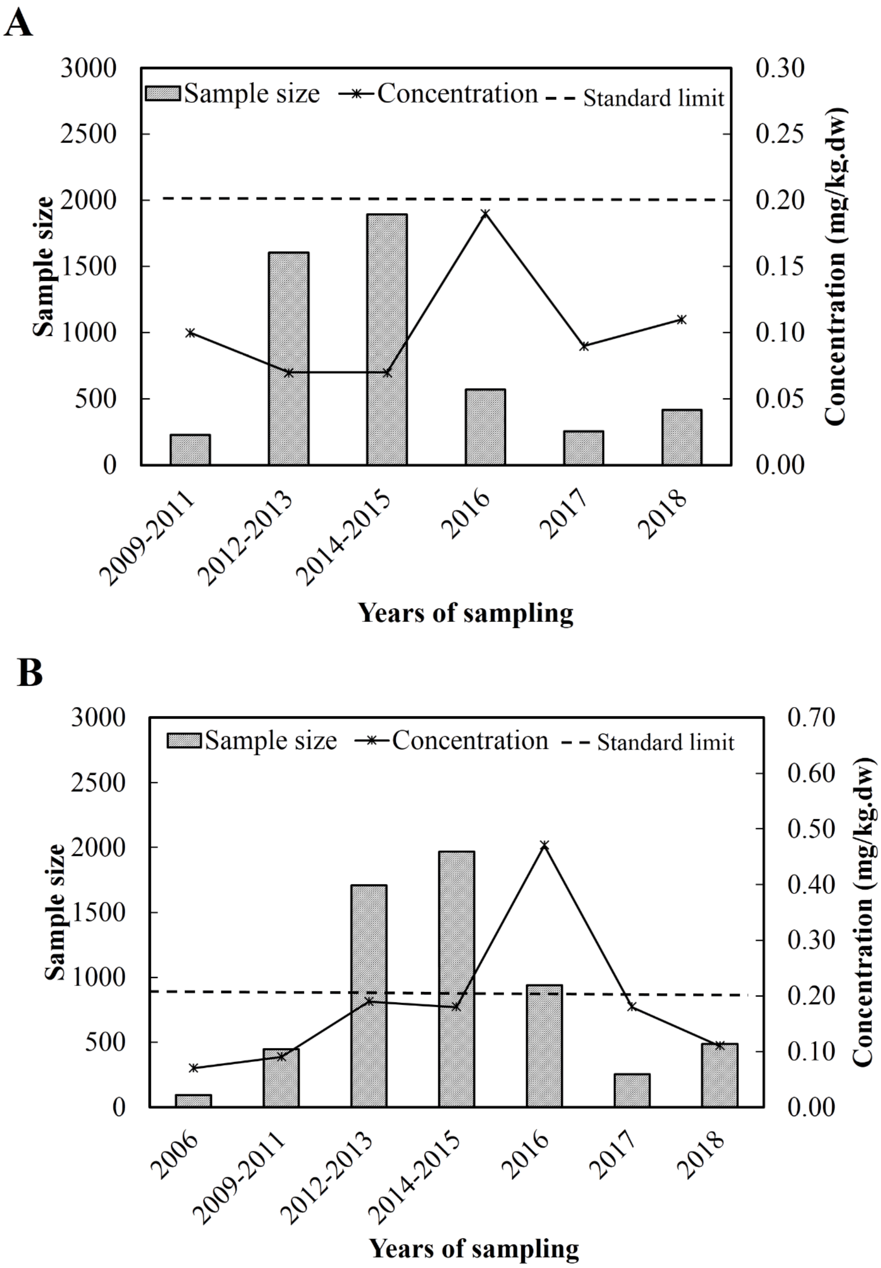
A
Sample size
Concentration
Standard limit
Sample size
Concentration (mg/kg.dw)
3000
2500
2000
1500
1000
500
0
0.30
0.25
0.20
0.15
0.10
0.05
0.00
2009-2011
2012-2013
2014-2015
2016
2017
2018
Years of sampling
B
Sample size
Concentration
Standard limit
Sample size
Concentration (mg/kg.dw)
3000
2500
2000
1500
1000
500
0
0.70
0.60
0.50
0.40
0.30
0.20
0.10
0.00
2006
2009-2011
2012-2013
2014-2015
2016
2017
2018
Years of sampling

Figure 6

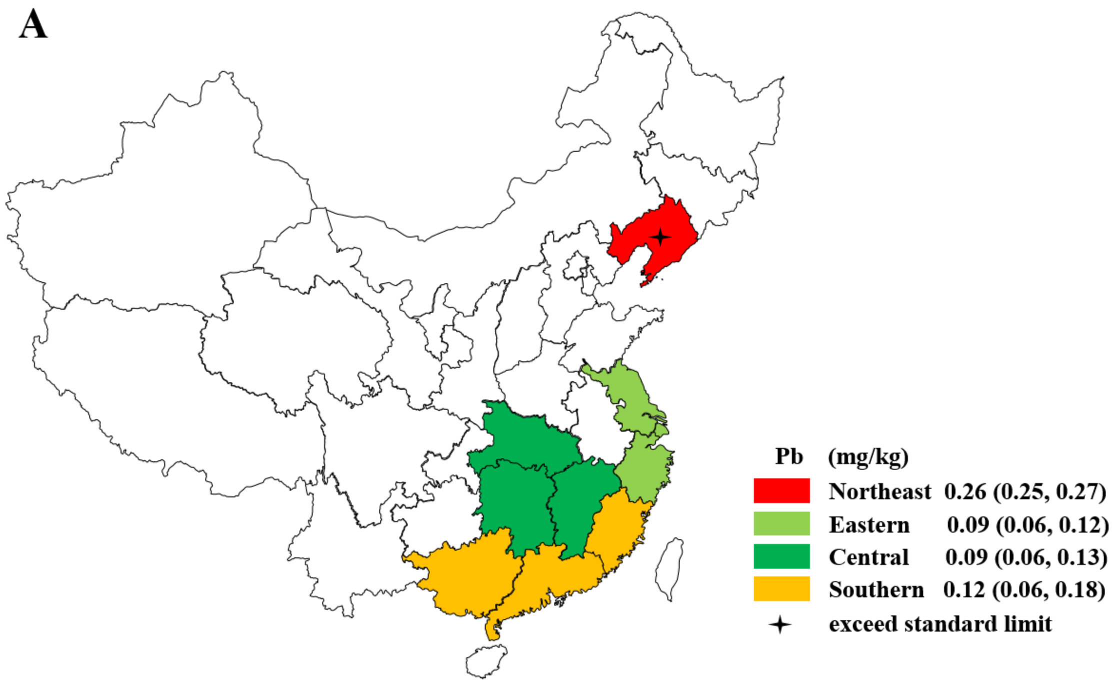
A
Pb (mg/kg)
Northeast 0.26 (0.25, 0.27)
Eastern 0.09 (0.06, 0.12)
Central 0.09 (0.06, 0.13)
Southern 0.12 (0.06, 0.18)
exceed standard limit

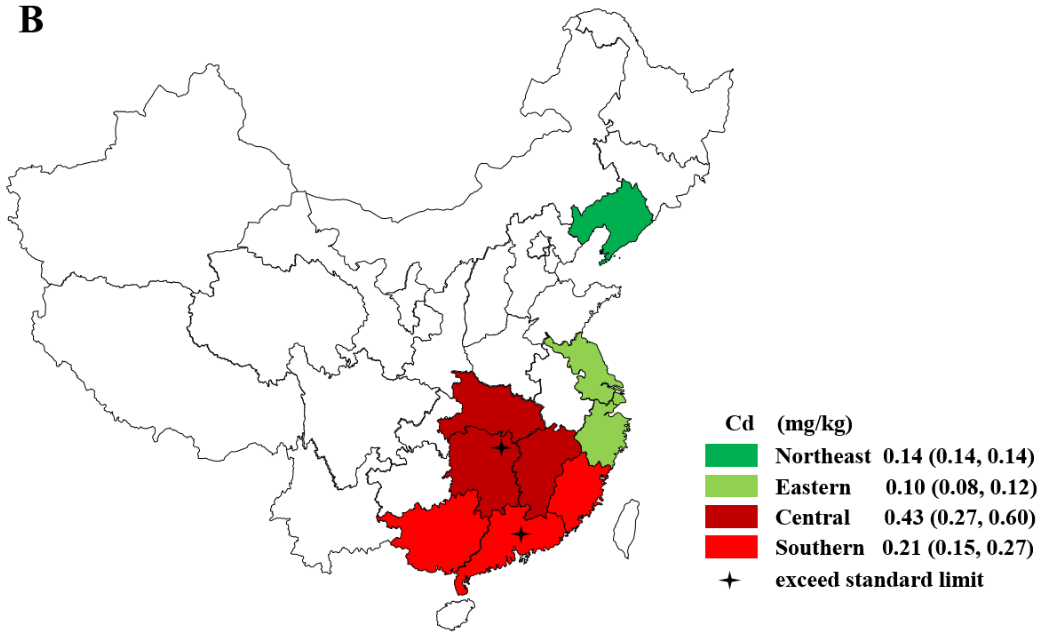
B
Cd (mg/kg)
Northeast 0.14 (0.14, 0.14)
Eastern 0.10 (0.08, 0.12)
Central 0.43 (0.27, 0.60)
Southern 0.21 (0.15, 0.27)
exceed standard limit

Click here to access/download
Supporting Information
Supplementary material for Publication.docx

Click here to access/download
Supporting Information
PRISMA_2020_checklist.docx
